# Supplementary material for: Multimodal activity-affinity assay of ADAM-10 extracellular vesicles in untreated plasma reveals metastatic stage of colorectal cancer
Source: Biosens Bioelectron. Author manuscript; Available in PMC 2026 Oct 7. (PMC13643713; doi:10.1016/j.bios.2026.118517)
Supplement: Supplementary file 1 [file NIHMS2210535-supplement-Supporting_Information.docx]

**Supporting Information**

Multimodal Activity-Affinity Assay of ADAM-10 Extracellular Vesicles in Untreated Plasma Reveals Metastatic Stage of Colorectal Cancer

Tiger Shi^1,†^, J. Alex Sinclair^1,†^, Youwen Zhang^1^, Xuemin Lu^2^, Sonu Kumar^1^, Xin Lu^2^, Satyajyoti Senapati^1^, and Hsueh-Chia Chang^1*^

^1^Department of Chemical and Biomolecular Engineering, University of Notre Dame, Indiana, 46556, USA.

^2^Department of Biological Sciences, University of Notre Dame, Indiana, 46556, USA.

^†^ Denotes Equal Author Contribution

**^*^Corresponding author:** [hchang@nd.edu](mailto:hchang@nd.edu)

**Supporting Fig. 1.** Translational Brownian motion tracking output for IJPs over the full 120s recording period. Each panel represent the 2D particle displacement trajectory of all individual particles within the xy measurement plane. Healthy patient examples are shown in the top row, pre-metastatic in the middle row, and metastatic in the bottom row. Recorded tracks are color-coded according to the video timestamp: dark green indicates early trajectory portions (0-10s), transitioning through intermediate yellow points, and concluding with late trajectory dark red points (110-120s). This temporal color grading reveals both spatial movement and frame entry/exit events, illustrating a comprehensive particle migration scheme across recording sections.

**Supporting Fig. 2.** ELISA protein assays for CD63 and ADAM-10. (a) ADAM-10 ELISA assay results for healthy human dermal (HHD) cell-cultured media fractioned per workflow in Figure 3a. CCM and FT fractions were tested in 10X concentrated using 10kDa MWCO centrifugal filter and 1X & 10D dilutions in 1X DPBS. Unlysed and lysed EV fractions were tested at 1X, 5D, and 25D dilutions in 1X DPBS. (b) Total ADAM-10 ELISA calibration curve prepared per manufacturer instruction, featuring linear log-log fit ($R^{2}$=0.9973) between standard ADAM-10 concentration and endpoint fluorescence assay after 20-min incubation with a limit of detection of 0.14 FI corresponding to 60 pg/mL total ADAM-10. (c) Total CD63 ELISA calibration curve prepared per manufacturer instruction, featuring a linear log-log fit ($R^{2}$=0.9975) between standard CD63 concentration and endpoint fluorescence assay after 20-min incubation with a limit of detection of 0.17 FI corresponding to 100 pg/mL total CD63. (e) Box plot of plasma concentration normalized CD63 concentration for each patient sample separated into each cohort. (f) Box plot of plasma concentration normalized ADAM-10 concentration for each patient sample separated into each cohort. Box plot significance values were determined using Welch’s t-Test.

**Supporting Fig. 3**. Comparison of diagnostic performance using Nile Red lipid fluorescence with bulk ADAM-10 activity rate (a-c), and a trimodal analysis which incorporates IJP period rotation (d-f). (a,d) Scatter plots illustrating the relationships Nile Red and ADAM-10 (a), and IJP period rotation (d), with groups colored by clinical group (healthy, pre-metastatic, metastatic). (b,e) Linear Discrimination Analysis (LDA) applied to scatter plots, with data-driven decision boundaries to separate the feature space into three regions, each corresponding to one of the three clinical groups. (c,f) Confusion matrices summarizing LDA classification performance for the bimodal (c) and trimodal (f) analyses. The bimodal Nile Red and ADAM-10 activity achieved an overall accuracy of 72.1%, while the trimodal model achieved 92.5%, which demonstrates a substantial improvement in discriminative capabilities when including the IJP-based characterization of ADAM-10+ EVs.

**Supporting Fig. 4.** Bulk ADAM-10 activity assays on additional cell lines. (a) Bulk ADAM-10 activity rates for healthy human dermal (HHD) and healthy mouse fibroblast (3T3) cell-cultured media fractioned per workflow in Figure 3a. (b) ADAM-10 bulk activity assay calibration curve prepared using serial dilutions of recombinant human ADAM-10 (rhADAM-10), featuring a linear log-log fit ($R^{2}$=0.9947) between rhADAM-10 concentration and fluorescent signal growth rate with a limit of detection signal of 20 FI/min corresponding to 4.8 ng/mL rhADAM-10. (c) Excitation & emission spectra of 5-FAM fluorophore measured at fixed wavelengths. To measure excitation, fixed emission wavelength was set to 525±20 nm. To measure emission, fixed excitation wavelength was set to 485±10 nm. (d) Normalized ADAM-10 activity per 108 EVs for four prostate cancer cell lines of increasing insensitivity to androgen: LNCaP, 22Rv1, DU145, and PC3. Higher androgen insensitivity in both cell culture media and isolated EVs suggest higher enzymatic activity correlates to disease progression.

**Supporting Fig. 5.** Additional information on Bradford total protein and Nile Red total lipid assays. (a) Nile Red fluorescent total lipid assay calibration curve prepared using serial dilutions of spin-filter isolated EVs from LNCaP and 22Rv1 cell cultured media, featuring a linear log-log fit ($R^{2}$=0.9960) between NTA-derived particle concentration and fluorescent intensities with a limit of detection of 130 FI corresponding to ${10}^{7}$ particles/mL. (b) Excitation & emission spectra of Nile Red lipid dye incubated with diluted healthy human plasma measured at fixed wavelengths. To measure excitation, fixed emission wavelength was set to 635$\pm$20 nm. To measure emission, fixed excitation wavelength was set to 555$\pm$10 nm. (c) Bradford colorimetric total protein assay calibration curve prepared using serial dilutions of bovine serum albumin (BSA), featuring a linear log-log fit ($R^{2}$=0.9880) between protein concentration and absorbance values with a limit of detection of 0.30 FI corresponding to 12 μg/mL total protein.
